# Availability of point-of-care culture and microscopy in general practice - does it lead to more appropriate use of antibiotics in patients with suspected urinary tract infection?

**DOI:** 10.1080/13814788.2020.1853697

**Published:** 2020-12-23

**Authors:** Anne Holm, Volkert Siersma, Lars Bjerrum, Gloria Cordoba

**Affiliations:** Research Unit for General Practice and Department of General Practice, University of Copenhagen Copenhagen, Denmark

**Keywords:** Urinary tract infections, microscopy, culture media, point-of-care testing, general practice

## Abstract

**Background:**

Urinary tract infection (UTI) is a common condition in general practice, and urine culture can help reduce inappropriate antibiotic prescriptions. In case of delay of the result, antibiotic treatment can be guided by one or more point-of-care (POC) tests. In Denmark, POC microscopy and POC urine culture are widely used for this purpose.

**Objectives:**

To investigate if availability of POC microscopy or POC culture in general practice was associated with a more appropriate treatment decision in patients with suspected UTI while waiting for the result from the microbiological laboratory.

**Methods:**

This prospective observational study was conducted in 2016 in general practice in the Copenhagen area, Denmark. Data on all patients presenting in general practice with symptoms of UTI were registered anonymously and a urine sample was sent for culture at the microbiological laboratory. The association between the availability of POC tests and the appropriateness of antibiotic prescribing was assessed with multivariable logistic regression.

**Results:**

Seventy-six general practices included 1545 patients (83% female); 71% received appropriate treatment in practices with POC culture available and 65% in practices without POC culture available (*p* = 0.042). Having a microscope available was not associated with more appropriate treatment (70% vs. 69%, *p* = 0.54).

**Conclusion:**

Availability of POC culture marginally increased appropriate treatment while waiting for the result from the microbiological laboratory. Practices should adopt a strategy where they either perform culture within the practice or send urine for culture at the microbiological laboratory.

**Trial registration number:**

ClinicalTrials.gov NCT02698332.

KEY MESSAGES

- In Danish general practice, availability of POC culture marginally increased appropriate antibiotic treatment while waiting for the result of urine sent to the microbiological laboratory.
- Availability of POC microscopy did not improve antibiotic treatment.
- The main reasons for inappropriate treatments were over- and undertreatment.

## Introduction

Urinary tract infection (UTI) is a common reason for consultation in general practice and the second-most frequent reason for antibiotic prescribing [1,2]. Overtreatment of UTI can cause antibiotic resistance without any clinical benefit for the patient. Undertreatment may lead to prolonged duration of symptoms or complications, such as acute pyelonephritis and septicaemia [3]. Most complications of UTI occur in the first week after consulting general practice [4].

Previous research has shown that the use of urine culture affects general practitioners’ (GPs’) antibiotic prescription for acute UTI [5]. Nonetheless, a quick response from the microbiological laboratory is essential to reduce inappropriate prescription of antibiotics [6,7]. In the Capital Region of Denmark, the response time of the microbiological laboratories varies between 1 and 4 days, which has been shown to increase overtreatment as the uncertainty about when the result arrives compromises a wait-and-see strategy [8].

In Denmark, point-of-care (POC) urine tests in general practice (urine dipsticks, POC microscopy and POC culture) are paid by fee for service, but it is up to the owner of the practice to decide if these POC tests should be available. At the time of conducting this study, there were no official guidelines on how to use the tests, and all practices can send samples to the microbiological laboratory. Almost all patients with suspected UTI in Denmark have a urine dipstick performed, about one-third have POC microscopy performed, two-thirds have POC culture performed and about one in four have urine sent to the microbiological laboratory [8].

Although using POC culture in the diagnostic pathway has been shown to improve antibiotic treatment of UTI, the use of POC tests can be influenced by numerous factors. A more reliable measure for authorities and investigators is whether having POC tests available in the practice can improve antibiotic treatment in a setting where culture at the microbiological department is available.

Availability of POC microscopy and POC culture could potentially provide a rapid diagnosis, avoiding over- or undertreatment while waiting for the result from the microbiological laboratory. Still, the opposite could also be the case depending on the use and interpretation of the tests.

We aimed to investigate whether availability of urine POC tests (microscopy and culture) was associated with a more appropriate treatment decision in patients with suspected UTI while waiting for the result from the microbiological laboratory. Urine dipstick testing could not be investigated because it is available in almost all practices.

## Methods

### Study design and setting

Prospective observational study in general practice embedded in a cluster randomised controlled trial. The practices in the original study (ClinicalTrials.gov: NCT02698332) were randomised to either receive a guideline on diagnosis of UTI or continue usual practice. The intervention turned out to not affect clinical practice or antibiotic prescription in any way because the practices did not use the guideline. Thus, the data were used as an observational data set on usual practice.

### Recruitment of general practices

Recruitment of general practices was done through online advertisement in email newsletters to general practice, invitation by post to 200 practices and invitation of 44 general practices already participating in a medical audit project about UTI [8]. Only practices in the Capital Region of Denmark were invited. The practices were offered a small remuneration and quality feedback on diagnosis and treatment of UTI in exchange for participation.

### Recruitment of patients

The practices were told to register diagnostics and treatment on the first 20–40 patients presenting in general practice with symptoms of UTI regardless of age, sex and comorbidity. Data collection was planned to take place in March–May 2016. Inclusion criteria were all patients presenting in general practice with any symptom that made the GP suspect UTI, and where urine was collected for investigation (i.e. not patients who were managed without the use of urine tests). Patients only had their first UTI within the study period registered. The only exclusion criterion was acute admission to the hospital.

### Data collection

Practices provided information about number of owners of the practice (GPs), other doctors (for example, GPs in training), number of nurses and other staff, patients attached to the practice and what diagnostics were available in the practice. Data collection from patients was performed prospectively and anonymously. The practices registered clinical data using a case report form designed following the Audit Project Odense (APO) methodology [9,10] (see Supplementary Appendix 1).

The data collection instrument aimed to secure consecutiveness for acute illnesses, as GPs can quickly fill in the required information.

On the day of consultation, clinical history, diagnostics, diagnosis and treatment were registered. All patients provided a urine sample, which was sent to the microbiological department. On the day after the consultation, the result of the POC urine culture, if such was performed, and the subsequent diagnosis and treatment were registered. GPs received answers of results of urine cultures performed at the hospital and registered results on a case report form to be used as the reference standard.

### Point-of-care tests

Practices could use the POC tests they had available in routine practice. They were asked to register if they had a microscope and a POC culture testing available but not which method they used. A wide variety of tests are in use. All available urine culture kits follow the same method: fresh urine is incubated directly on the agar and the result is read on the following day. Microscopy is performed on fresh urine and the result can be read immediately. The majority uses Flexicult SSI Urinary Kit^TM^ for culture and a phase-contrast microscope for microscopy (unpublished data from a previous audit [8]). Flexicult SSI Urinary Kit^TM^ has previously shown to have a high sensitivity (0.86) and low specificity (0.54) [11]. Microscopy has been shown to have varying diagnostic performance but has not been studied recently in the Danish context [12].

### Culture at the microbiological laboratory – Reference test

Urine for the microbiological department was incubated in a standardised boric acid container and transported to the microbiological laboratories (Herlev and Hvidovre). Significant growth was defined as growth of ≥10^3^ cfu/mL for *Escherishia coli* and *Staphylococcus saprofyticus*, ≥10^4^ cfu/mL for other typical uropathogens and ≥10^5^ cfu/mL for possible uropathogens per European consensus [13]. Plates with significant growth of more than two uropathogens were labelled as mixed cultures (inconclusive). Inconclusive cultures were classified as negatives for analysis. The susceptibility pattern was determined for mecillinam, trimethoprim, nitrofurantoin and sulfamethizol, among others.

### Outcome and variables

The primary outcome was appropriate treatment decision on the day after consultation defined as either (1) having UTI according to the reference and receiving a first-line antibiotic to which the infecting pathogen was susceptible, (2) having UTI according to the reference and receiving a second-line antibiotic to which the infecting pathogen was susceptible, if the patient was allergic or the infecting pathogen was resistant to all first-line antibiotics, or (3) not having UTI according to the reference and not receiving an antibiotic. First-line antibiotics were defined according to Danish recommendations in 2016 as either pivmecillinam, sulfamethizole, trimethoprim or nitrofurantoin [14].

The investigated variables were: (1) availability of POC microscopy in general practice and (2) availability of POC culture in general practice. Availability was chosen instead of use because the availability of POC tests is not affected by individual patient factors or previous test results.

Covariates used for confounder adjustment were: number of patients listed to the practice, number of owners of the practice, number of doctors employed, number of nurses employed, other staff engaged, recent participation in audit regarding UTI, patient age, and patient sex. Patient age and sex are indicators for the case mix of patients in the practice. Other factors that may be related to appropriate treatment (i.e. symptoms and use of POC tests) were considered intermediate variables and therefore were not considered for confounder adjustment.

### Sample size calculation and statistical analysis

Assuming 70% are appropriately treated in practices where POC culture is not available, and taking clustering into account, to detect a 10 percentage-point difference in the outcome, we would need 50 practices to recruit 18 patients each for 80% power with a 5% significance level. Appropriate prescribing was analysed in multivariable logistic regression models that account for practice clustering through generalised estimating equations (GEE). Differences in distribution of baseline data between practices with and without POC culture and microscopy were investigated using chi-squared tests or *t*-tests for practice factors, and a logistic regression model using GEE to account for practice clustering for patient factors. Statistical analysis was performed using SAS 9.4.

### Ethics and data protection

The study was presented to the ethical committee of Copenhagen and did not require ethical approval (ref. H-15015686). The Danish Health Legislation Act requires patients to consent to all diagnostics and treatment, including the additional urine sent for culture. Patient data were anonymised before being sent from the GP to the investigators and did not, therefore, require approval from the Danish data protection agency.

## Results

A total of 90 practices accepted participation in the study and 76 practices completed inclusion of 1545 patients between 1 March and 7 June 2016. Data collection was planned to finish 31 May 2016, but some of the practices included patients for an additional seven days because they had a late start.

**Figure 1.**
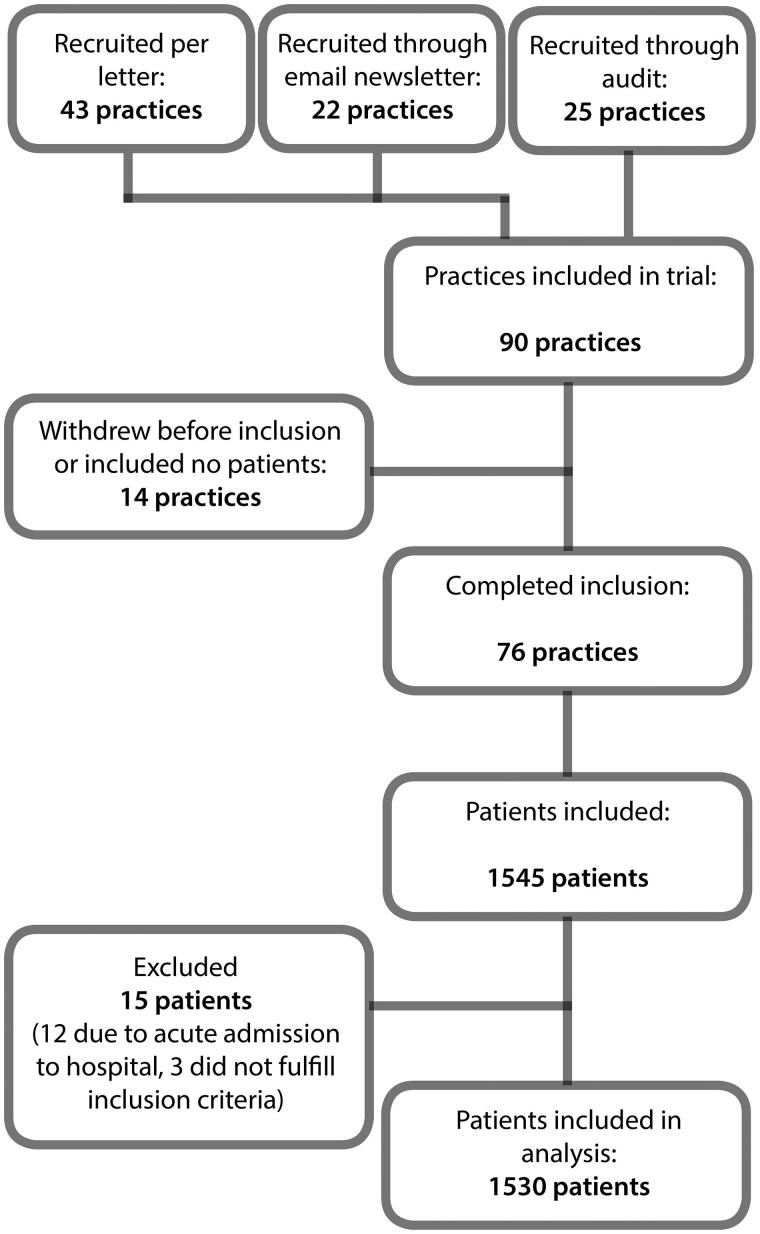
Inclusion flow chart.

Fifteen patients had to be excluded, leaving 1530 patients with symptoms of suspected UTI included in the analyses (see Figure 1). Practices that withdrew from the project or did not include patients did not differ significantly from those who completed inclusion.

Baseline data for practices are shown in Table 1. Practices with microscope and POC culture available were generally larger than those without. Practices with a microscope had significantly more patients (*p* = 0.03), more owners (*p* = 0.006) and more employed doctors (*p* = 0.03).

**Table 1.** Baseline data for participating practices (*n* = 76).

	All *N* = 76	Microscope available, *N* = 27 (36% of practices)	Culture available, *N* = 58^b^ (76% of practices)
Practice size			
Small practice (0–1999 patients) (%)^a^	26 (35)	6 (22)	19 (33)
Medium-sized practice (2000–3999 patients) (%)^a^	34 (45)	11 (41)	25 (44)
Large practice (4000 or more patients) (%)^a^	15 (20)	10 (37)	13 (23)
Practice type			
Solo practice (%)	37 (49)	10 (37)	24 (41)
2–3 owners (%)	31 (41)	9 (33)	28 (48)
4 or more owners (%)	8 (11)	8 (30)	6 (10)
Number of doctors			
No employed doctors (%)	27 (36)	8 (30)	23 (40)
1 employed doctors (%)	35 (46)	13 (48)	26 (45)
2 or more employed doctors (%)	14 (18)	6 (22)	9 (16)
Number of nurses			
No employed nurses (%)	16 (21)	7 (26)	12 (21)
1 employed nurse (%)	37 (49)	8 (30)	25 (43)
2 or more employed nurses (%)	23 (30)	12 (44)	21 (36)
Number of other staff			
No other staff (%)	11 (14)	2 (7)	9 (16)
1 other staff (%)	21 (28)	6 (22)	17 (29)
2 or more other staff (%)	44 (58)	19 (70)	32 (55)
Recent participation in audit (%)	20 (26)	11 (41)	16 (28)

POC, point-of-care.

^a^Missing data on practice size = 1.

^b^*N* = 57 for practice size.

The 1530 included patients were predominantly female (83%). Almost everyone had a dipstick performed, 21% had POC microscopy performed and 63% had POC culture performed; 46% had confirmed UTI in the reference test. Patient characteristics did not differ significantly between groups (Table 2).

**Table 2.** Baseline data for included patients (*n* = 1530).

	All, *N* = 1530	Microscope available, *N* = 607 (40%)	Culture available, *N* = 1205 (79%)
Age			
Age 0–29 (%)	335 (22)	130 (21)	248 (21)
Age 30–59 years (%)	501 (33)	205 (34)	384 (32)
Age 60 years or more (%)	694 (45)	272 (45)	573 (48)
Women (%)^a^	1268 (83)	505 (84)	995 (83)
Urine dipstick performed (%)	1489 (97)	575 (95)	1169 (97)
Microscopy performed (%)	323 (21)	323 (53)	255 (21)
POC culture performed (%)	957 (63)	363 (60)	952 (79)
Significant growth in reference (%)^b^	692 (47)	280 (48)	536 (46)

POC, point-of-care.

^a^Missing data on sex = 4.

^b^Missing data on reference result = 53.

**Figure 2.**
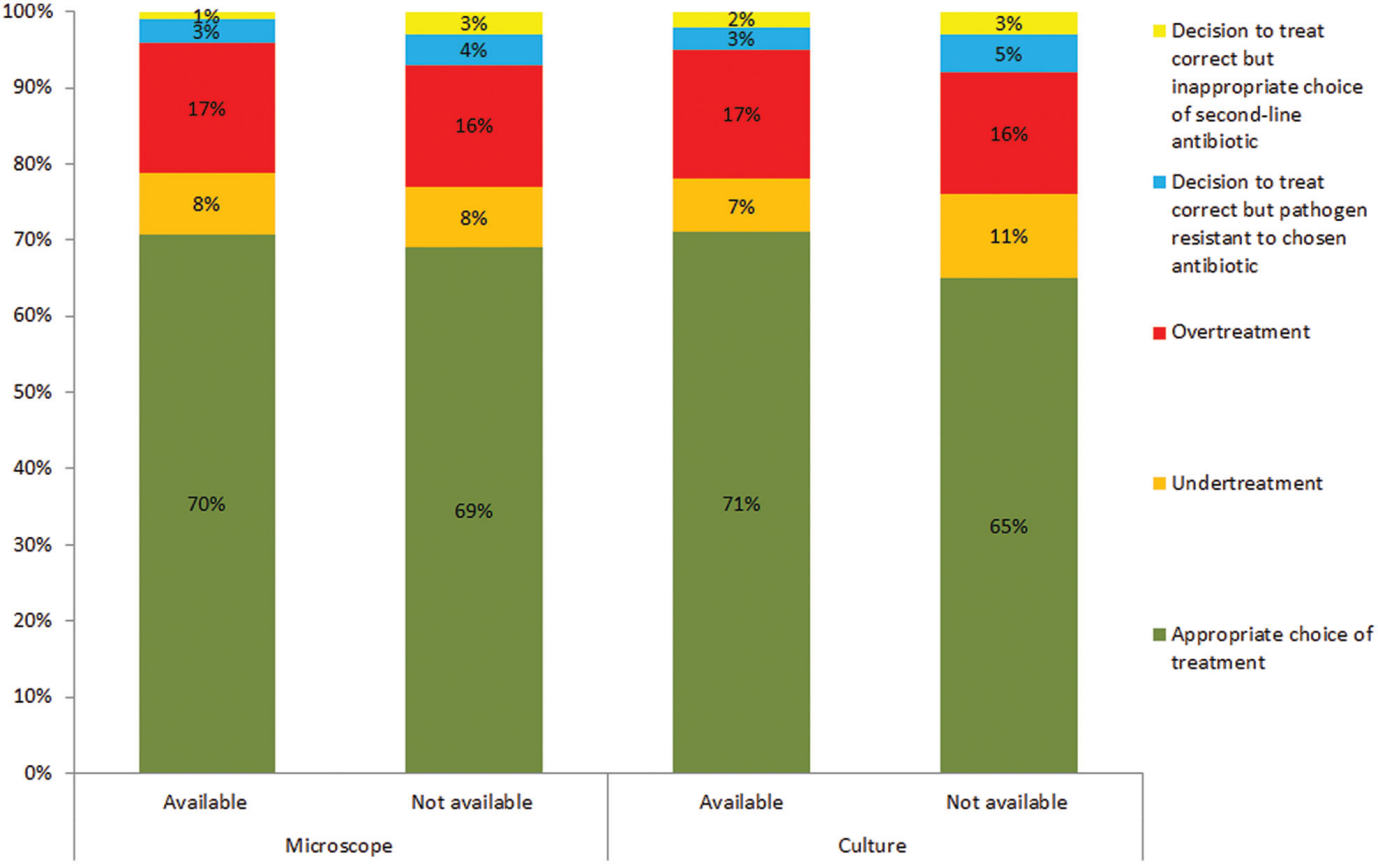
Proportion of patients treated appropriately or inappropriately the day after consultation and reasons for inappropriate treatment in practices with and without point-of-care tests available.

Table 3 shows the primary and secondary outcomes. Having POC culture available was significantly associated with more appropriate treatment. Having a microscope in the practice was not associated with more appropriate treatment. In practices with POC culture available, 71% of patients received appropriate treatment on the day after consultation compared to 65% in practices without POC culture. Inappropriate treatment was primarily due to overtreatment (16%–19%) and undertreatment (6%–11%) (Figure 2).

**Table 3.** Odds for receiving appropriate antibiotic treatment the day after consultation if the patient is diagnosed in a practice with the point-of-care (POC) test available compared to patients diagnosed in practices without the POC test available.

	OR unadjusted	*p*	OR adjusted	*p*
Microscopy	1.08 (0.83–1.41)	0.54	1.09 (0.78–1.53)	0.600
Culture	1.29 (1.01–1.64)	0.042	1.36 (1.01–1.83)	0.046

Covariates used for confounder adjustment were: number of patients listed to the practice, number of owners of the practice, number of doctors employed, number of nurses employed, other staff employed, recent participation in audit regarding UTI, patient age, and patient sex.

## Discussion

### Main findings

In this study, we found that POC culture availability in general practice was associated with more appropriate antibiotic treatment the day after consultation when a reference culture was performed simultaneously at the microbiological laboratory. However, the difference between groups was less than the 10 percentage-points we had deemed clinically significant. Availability of a microscope in the practice was not seen to be associated with more appropriate antibiotic treatment.

### Strengths and limitations

This study was an extensive audit including more than 10% of practices in the Capital Region of Denmark. The audit was sufficiently simple to allow the participating practices to include on average 20 patients per practice resulting in a large sample of included patients. The simplicity of the study, however, had the drawback that few variables were registered for each patient, so we may have overlooked possible indicators of the case mix in the practice (i.e. comorbidities and pregnancy).

Our included practices were much larger than the average general practice in the area [15]. Participating practices could also be presumed to have POC culture and microscopy available more often than the average because this could serve as a motivation for participation. However, we did manage to include a sufficient number of practices without POC culture and microscopy to test our hypothesis. The validity of our findings is supported by the fact that microscopy was not associated with the outcome, although practices investing in a microscope could also be assumed to be more interested in UTI than the average general practice.

We detected a significant effect of having POC culture available in the practice when also performing culture at the microbiological laboratory, but the difference was small. This could be due to various factors: (1) GPs are already aware of restrictive use of antibiotics and withhold treatment more often than expected, (2) practices without POC culture delayed treatment and waited for the result of the reference test, and (3) practices with POC culture available differ from practices without POC culture available in aspects we were not able to control for.

### Interpretation of results in relation to existing literature

A recent observational study with an external reference culture showed that using POC culture in the diagnostic process could reduce overtreatment compared to when POC culture was not used [8]. We were not able to find any significant reduction in overtreatment in our study, but this may have been due to the reference standard being made available to the GPs as described above.

A Danish study, which randomised patients to either POC culture or POC culture and susceptibility testing, found appropriate treatment rates of 75% and 67% in the groups, respectively [16]. These results are quite similar to ours and it must be assumed that practices can obtain around 70% appropriate treatment by having POC culture available, possibly higher if POC culture is applied and interpreted appropriately.

## Conclusion

POC culture and microscopy have no clinically relevant effect on appropriate prescription of antibiotics when urine is also sent to the microbiological department for culture. Practices should adopt a strategy where they either perform POC culture within the practice or send urine for culture to the microbiological laboratory.

## Supplementary Material

Appendix 1: Registration Form
