# Supplementary figures and images for: Availability of point-of-care culture and microscopy in general practice - does it lead to more appropriate use of antibiotics in patients with suspected urinary tract infection?
Source: Eur J Gen Pract. 2020 Dec 23;26(1):175–81. doi: 10.1080/13814788.2020.1853697 (PMC7781897; doi:10.1080/13814788.2020.1853697)

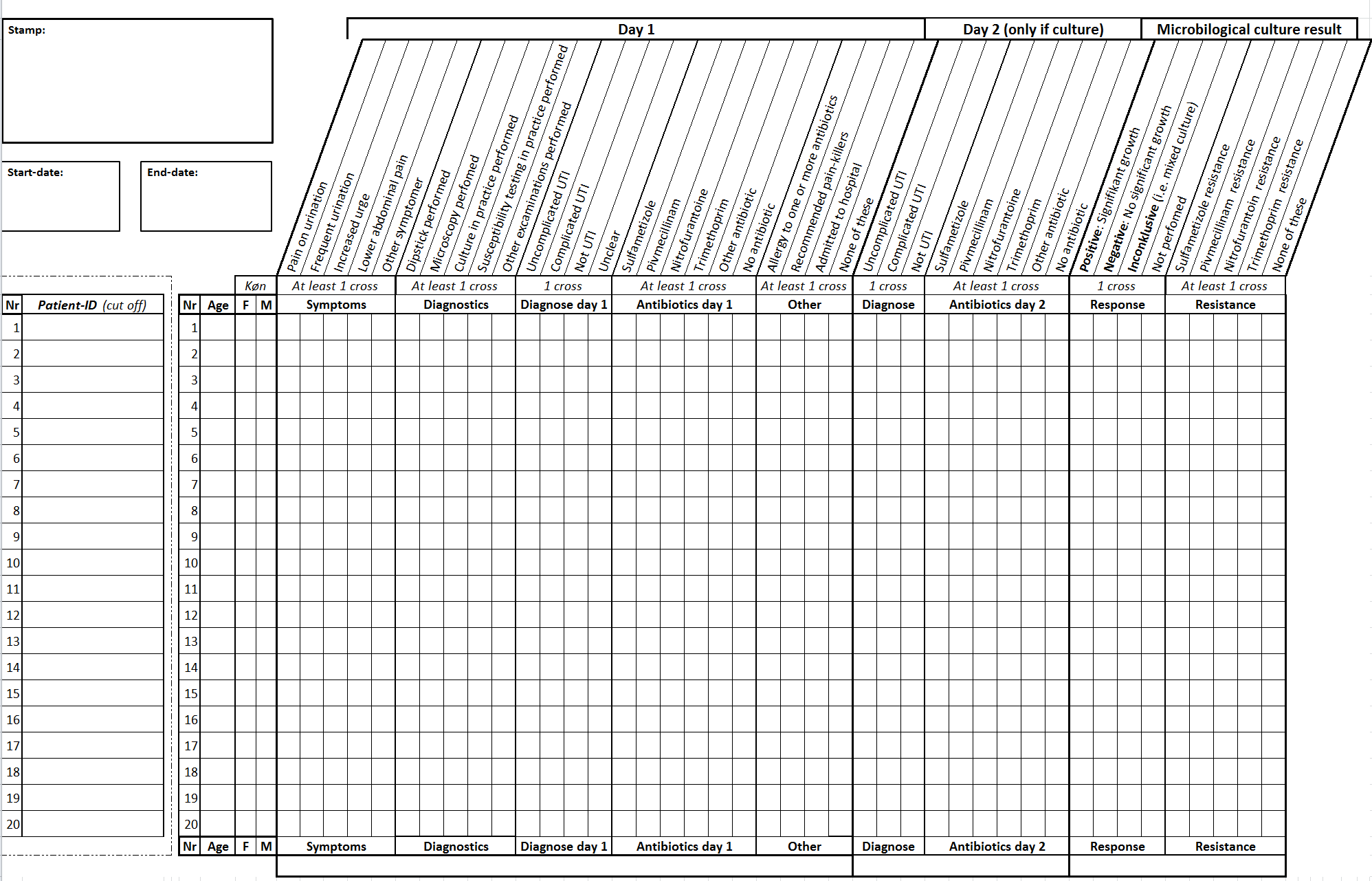

Supplement: Appendix 1: Registration Form [file IGEN_A_1853697_SM6568.png]
